# Parastomal Varices as an Atypical Source of Bleeding From a Urostomy in a Patient With Alcoholic Liver Disease

**DOI:** 10.7759/cureus.73886

**Published:** 2024-11-18

**Authors:** Omar Desouky, Baraa Chkir, Thihnaan Zuhair

**Affiliations:** 1 Urology, Royal Preston Hospital, Lancashire Teaching Hospitals NHS Foundation Trust, Preston, GBR; 2 Urology, Furness General Hospital, Barrow-in-Furness, GBR; 3 Internal Medicine, North Manchester General Hospital, Manchester University NHS Foundation Trust, Manchester, GBR

**Keywords:** alcoholic liver diseases, bleeding varices, portal hypertension, stoma, varices

## Abstract

Parastomal varices are an uncommon but significant source of hemorrhage in patients with portal hypertension, often posing diagnostic and therapeutic challenges. We report the case of a 73-year-old male with a history of alcoholic liver disease and a urostomy following cystoprostatectomy for bladder cancer. The patient presented with profuse bleeding from his urostomy site. Imaging revealed dilated vessels within the ileal conduit and associated mesentery and peri esophageal varices, suggesting possible underlying portal hypertension. This case highlights the importance of considering parastomal varices in patients with stomas and liver disease who present with bleeding.

## Introduction

Portal hypertension in chronic liver diseases, such as alcoholic liver disease, results from increased resistance to portal blood flow and leads to the development of collateral circulation [1]. Variceal bleeding is a well-recognized manifestation of portal hypertension, typically occurring in the esophagus and stomach [2]. However, ectopic varices, including parastomal varices, represent a rare but significant source of hemorrhage in patients with portal hypertension [3-5].

Parastomal varices are dilated venous collaterals that develop around stoma sites as a result of portosystemic shunts formed between the mesenteric veins of the ileal conduit and the subcutaneous circulation [3-5]. This abnormal vascular connection arises in response to increased portal venous pressure, often associated with portal hypertension, allowing blood to bypass the liver and circulate through alternative pathways [3-5]. While bleeding from parastomal varices is uncommon, it can be life-threatening and poses diagnostic and therapeutic challenges [4,5].

Ectopic varices are an uncommon source of gastrointestinal bleeding, accounting for up to 5% of variceal hemorrhages [6]. In a review of 169 cases of bleeding ectopic varices, 17% were located in the duodenum, 17% in the jejunum or ileum, 14% in the colon, 8% in the rectum, and 9% in the peritoneum [6]. Peristomal varices accounted for 26% of cases, with a few occurring at other rare sites [6].

Recognition of parastomal variceal bleeding is critical, especially in patients with undiagnosed portal hypertension, as timely management can prevent significant morbidity and mortality [4,5]. We present a case of a 73-year-old male with known liver disease with undiagnosed portal hypertension and a urostomy who presented with significant bleeding from his stoma, highlighting the importance of considering parastomal varices as a potential source of bleeding in such patients.

## Case presentation

A 73-year-old male presented to the emergency department with significant bleeding from his urostomy site first noticed around 2 a.m. The bleeding appeared to originate from the junction between the skin and the stoma, initially presenting as a mixture of blood and urine but soon progressing to a larger volume of dark red blood. Over the next several hours, the bleeding intensified, producing clots and necessitating frequent changes of the urostomy bag - six times in total due to the volume of blood. At one point, the bleeding became forceful, reportedly spurting from the stoma and hitting the wall, indicating a high-pressure bleed. Consent for publication was obtained from the patient during the admission. 

The patient’s past medical history included bladder cancer, for which he underwent a cystoprostatectomy with ileal conduit diversion in 2015. Following surgery, he developed recurrent parastomal hernias, with three unsuccessful repair attempts. Additionally, he had a history of alcoholic liver disease, diagnosed in 2016, along with other comorbid conditions including stage 3 chronic kidney disease (CKD), and a transient ischemic attack (TIA), he was commenced on clopidogrel following that.

In 2016, the patient was diagnosed with alcoholic liver disease after presenting with abdominal pain and ascites. His ascites were managed with drainage, and he was discharged on spironolactone and furosemide. An ultrasound scan at the time showed liver coarsening. Despite counseling on the importance of alcohol abstinence, he initially ceased drinking but experienced multiple relapses over the following years. During this admission, he reported consuming two to three pints of beer per week.

Upon examination, the patient was hemodynamically stable with a blood pressure of 117/71. Remaining vitals were within normal range. His abdomen was soft, and the urostomy appeared healthy with no active bleeding at the time of assessment. There were obvious venous collaterals seen surrounding the urostomy. The patient noted the bleeding to have originated superficially from the skin edge. There was a soft and reducible long-standing parastomal hernia. Blood tests taken earlier in the morning of admission indicated a hemoglobin level of 87 g/L, the most recent result previous to that was from 2020 with a hemoglobin level of 123 g/L. Coagulation studies were normal with an international normalized ratio (INR) of 1. The model for end-stage liver disease (MELD) score was 11, suggesting a 6.0% estimated three-month mortality. Other lab results on admission are shown in Table 1.

**Table 1:** Initial lab test results

Lab tests	Results	Normal values
Hemoglobin	87 g/L	38–172 g/L
Platelets	202 x 10^9^/L	150-450 x 10^9^/L
Gamma-glutamyl transferase (GGT)	328 U/L	0 to 50 U/L
Albumin	30 g/L	34 to 54 g/L
Alanine aminotransferase (ALT)	27 U/L	0 to 45 U/L
Alkaline phosphatase (ALP)	111 U/L	30–130 U/L

**Figure 1:**
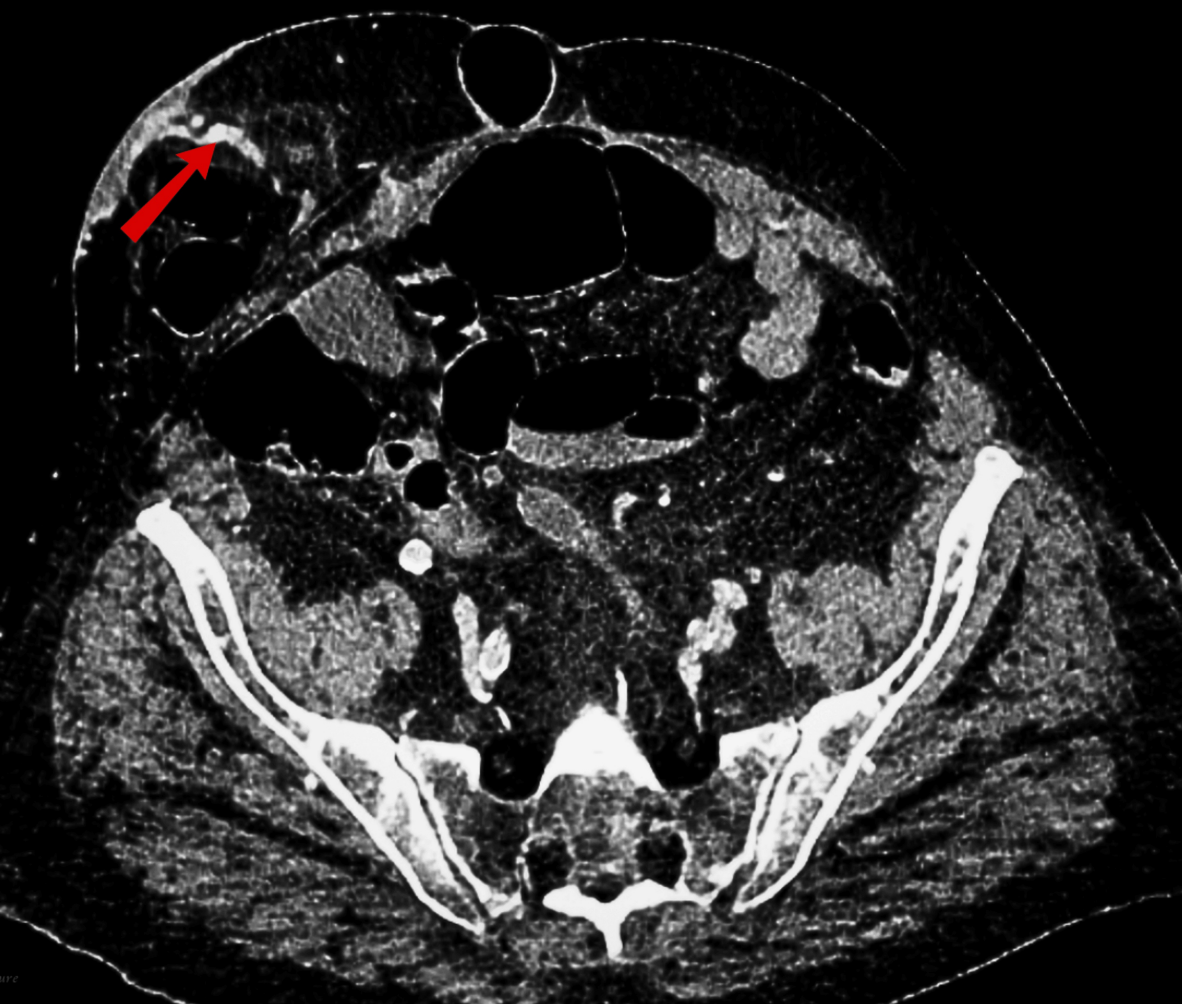
CT scan showing abnormal dilated vein arising from the conduit

The initial management plan included holding the patient’s clopidogrel, administering intravenous fluids, and closely monitoring the patient. The bleeding settled immediately after admission; regular monitoring was done by examining the ileostomy bag contents to ensure no further episodes of bleeding. The gastroenterology team was consulted due to the suspected involvement of portal hypertension in the bleeding, given the patient’s history of alcoholic liver disease. They advised that, given the patient's stable condition, conservative management with beta-blockers could be considered if portal hypertension is confirmed as the underlying cause. A computed tomography (CT) scan revealed dilated vessels within the ileal conduit and mesentery as well as perioesophageal varices, findings suggestive of portal hypertension (Figure 1). Furthermore, the scan demonstrated that the liver generally has low attenuation, consistent with fatty infiltration. Four days after admission there were still no further bleeding episodes. The patient was subsequently started on carvedilol, 12.5 milligrams (mg) once daily, to reduce portal pressure and referred for follow-up with the gastroenterology team as well as an endoscopy.

## Discussion

This case illustrates a rare presentation of parastomal variceal bleeding in a patient with alcoholic liver disease and previously undiagnosed portal hypertension. The development of parastomal varices results from increased portal venous pressure leading to the formation of collateral vessels at the stoma site [4,5]. In patients with stomas, the abdominal wall becomes a potential site for portosystemic shunting due to the disruption of normal vascular pathways [4,5].

Bleeding from parastomal varices is a serious complication that can lead to increased morbidity and mortality [7]. In this case, the initial presentation with significant bleeding and the presence of dilated vessels on imaging pointed towards parastomal varices associated with portal hypertension.

Management options for parastomal variceal bleeding include conservative measures, pharmacological therapy, endoscopic interventions, interventional radiology procedures, and surgical approaches [8]. Non-selective beta-blockers (NSBB), such as carvedilol, are used to reduce portal pressure and have demonstrated superior efficacy in managing portal hypertension compared to traditional NSBBs [9,10]. Carvedilol not only reduces hyperdynamic circulation and splanchnic vasodilation but also decreases intrahepatic resistance, making it particularly effective for clinically significant portal hypertension [10].

In refractory cases, transjugular intrahepatic portosystemic shunt (TIPS) placement or surgical revision of the stoma may be considered [11]. Liver transplantation in surgically fit patients has a crucial role in managing portal hypertension [12].

Preventive strategies are essential for patients with known alcoholic liver disease, including regular monitoring for signs of portal hypertension and educating patients to recognize bleeding symptoms, which can enable timely intervention [13,14]. This case highlights the need for a high index of suspicion for parastomal varices in patients with stomas and liver disease presenting with bleeding.

## Conclusions

Parastomal variceal bleeding is a rare but life-threatening complication in patients with portal hypertension and stomas. This case emphasizes the necessity for clinicians to maintain a high index of suspicion for parastomal varices when encountering bleeding at stoma sites, especially in patients with known or suspected liver disease. Early recognition through imaging and collaboration with the multidisciplinary team is crucial for effective management. Follow-up to assess the patient’s condition, including monitoring for re-bleeding after intervention, is crucial for timely treatment adjustments and optimizing outcomes. 
